# Preoperative sarcopenia negatively impacts short‐ and long‐term outcomes of rectal cancer: A propensity score‐matched analysis

**DOI:** 10.1002/ags3.12889

**Published:** 2024-11-24

**Authors:** Shinya Abe, Hiroaki Nozawa, Kazuhito Sasaki, Koji Murono, Shigenobu Emoto, Kensuke Kaneko, Yuichiro Yokoyama, Hiroyuki Matsuzaki, Yuzo Nagai, Soichiro Ishihara

**Affiliations:** ^1^ Department of Surgical Oncology Graduate School of Medicine, The University of Tokyo Bunkyo‐ku Tokyo Japan

**Keywords:** outcome, propensity score matching, rectal cancer, sarcopenia

## Abstract

**Aim:**

Sarcopenia is associated with poor postoperative outcomes in various cancers; however, limited evidence is available for rectal cancer. Therefore, the present study examined the effects of skeletal muscle mass on the short‐ and long‐term outcomes of rectal cancer.

**Materials and Methods:**

A total of 787 Stage I–IV rectal cancer patients who underwent curative resection between 2003 and 2021 at The University of Tokyo Hospital were included. We conducted a propensity score‐matched analysis to mitigate confounding bias. The third lumber psoas muscle mass was measured to define sarcopenia.

**Results:**

Among 787 patients, 350 (44.5%) were classified as having sarcopenia. After matching, 532 patients were analyzed. Patient characteristics in the sarcopenia and nonsarcopenia groups were similar; however, the body mass index differed. The sarcopenia group had significantly higher rates of postoperative complications of all grades (33.1% vs 24.8%; *p* = 0.035), of grade ≥2 (29.3% vs 21.8%; *p* = 0.047), and anastomotic leakage (1.9% vs 0%; *p* = 0.0082) than the nonsarcopenia group. The 5‐y overall survival rate was significantly lower in the sarcopenia group than in the nonsarcopenia group (85.3% vs 91.8%, *p* = 0.019). Disease‐free survival was similar between the groups (*p* = 0.40). In the total cohort analysis, sarcopenia was an independent risk factor for total postoperative complications (odds ratio 1.41, *p* = 0.042).

**Conclusion:**

Preoperative sarcopenia is associated with more total postoperative complications, more anastomotic leakage, and worse survival in rectal cancer patients.

## Graphical abstract

This study examined the effects of skeletal muscle mass on the short‐ and long‐term outcomes of rectal cancer. This study revealed that preoperative sarcopenia was associated with more total postoperative complications, more anastomotic leakage, and worse survival in rectal cancer patients.

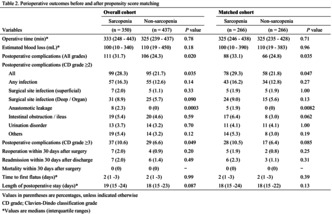

## 1 INTRODUCTION

Sarcopenia was reported by Rosenberg in 1989 as an age‐related loss of skeletal muscle mass. However, the definition of sarcopenia has since changed, with several working groups on sarcopenia proposing that it is characterized by a decline in muscle mass and function.^2^ In clinical practice in the oncology field, computed tomography (CT) is a routine part of imaging examinations to diagnose cancer progression and select a treatment strategy. Therefore, muscle mass on pretreatment CT images is used in analyses. Several meta‐analyses reported that sarcopenia, defined using preoperative CT images, was associated with postoperative complications in various cancers. Tajero‐Avila et al showed that sarcopenia was related to postoperative complications and a prolonged hospital stay in colorectal cancer patients, but not in rectal cancer patients.^3^ To the best of our knowledge, only two studies demonstrated that sarcopenia was associated with postoperative complications in rectal cancer patients. One study identified sarcopenia as a more accurate predictive factor for postoperative complications in a relatively small cohort,^4^ while the other found that sarcopenia was related to postoperative remote infection, but did not observe a relationship between sarcopenia and total postoperative complications.^5^ These findings may be attributed to the more pronounced effects of patient‐ and cancer‐related factors besides sarcopenia in rectal surgery than in colon surgery.

Rectal cancer surgery demands higher technical skills than colon cancer surgery due to a number of clinical and anatomical factors, such as a narrow pelvic space, high body mass index (BMI), and bulky mesorectum.^6^, ^7^ Therefore, the rate of postoperative complications is higher for rectal cancer surgery than for colon cancer surgery.

The present study examined short‐ and long‐term outcomes associated with sarcopenia for rectal cancer using a propensity score‐matched (PSM) analysis to mitigate confounding bias.

## 2 METHODS

### 2.1 Patients

**FIGURE 1:**
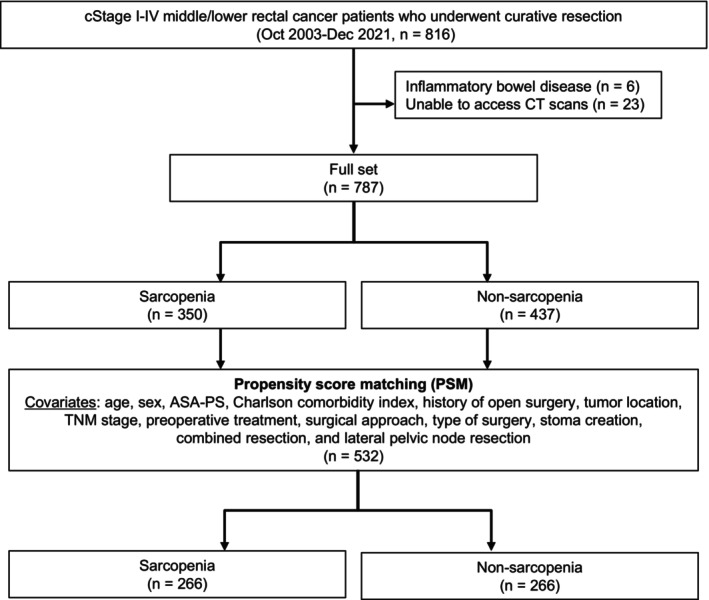
Study cohort selection process. Eight hundred and sixteen patients with clinical Stage I–IV rectal cancer who underwent radical surgery were enrolled between October 2003 and December 2021. Seven hundred and eighty‐seven patients were included in the final study population. Five hundred and thirty‐two patients were analyzed after propensity score matching.

We enrolled consecutive patients with clinical Stage I–IV middle/lower rectal cancer who underwent curative resection between October 2003 and December 2021 at The University of Tokyo Hospital. Among 816 patients, those with inflammatory bowel disease or preoperative computed tomography (CT) images that were inaccessible were excluded. Therefore, 787 patients were analyzed in this retrospective single‐institutional study (Figure 1). The present study was approved by the Institutional Ethics Committee of The University of Tokyo (No. 3252‐[15]) and informed consent was obtained through an opt‐out method.

### 2.2 Treatment strategy

Regarding preoperative treatment, indications were based on pretreatment cancer staging by colonoscopy, CT, magnetic resonance imaging, and positron emission tomography/CT (PET/CT). Patients with locally advanced tumors (cT3‐4/any N/M0 or any T/N+/M0) below the peritoneal reflection or a rectal tumor reaching the peritoneal reflection in a preoperative examination received preoperative chemoradiotherapy (CRT) with radiation (55 Gy in 25 fractions or 50.4 Gy in 28 fractions for 5 weeks) and mainly 5‐fluorouracil‐based oral administration. Total mesorectal excision (TME) was generally performed 6–10 weeks after the completion of CRT. Lateral pelvic lymph node dissection (LPND) was selected when lymph nodes of a longitudinal diameter ≥8 mm were detected on CT before pretreatment imaging investigations (selective LPND). Preoperative treatment, including CRT or chemotherapy for patients with distant metastasis, was selectively conducted. All resected specimens after surgery were pathologically analyzed. Pathological staging was performed according to the TNM classification system. Adjuvant chemotherapy (ACT) was considered for all patients based on pathological staging according to the Japanese Society for Cancer of the Colon and Rectum guidelines.^8^ The final decision to administer ACT was individually customized based on discussions between clinicians and patients.

### 2.3 Outcome measures

Postoperative complications occurring within 30 d after surgery were defined based on the Clavien–Dindo classification system (CD). Anastomotic leakage was defined as symptomatic leakage, which require therapeutic intervention, such as draining, antibiotic treatments, and surgical intervention.^9^ Overall survival (OS) was defined as the interval between the date of surgery and death or the last follow‐up. Disease‐free survival (DFS) was defined as the interval between the date of surgery and death, recurrence, the detection of malignant disease, or the last follow‐up. Postoperative surveillance was conducted according to the recommendations of the surveillance protocol in the Japanese Society for Cancer of the Colon and Rectum guidelines.^8^ This protocol involves an investigation of serum carcinoembryonic antigen levels, performing CT scans, and conducting colonoscopy for 5 y after surgery.

### 2.4 Sarcopenia

All patients underwent preoperative CT, which was used to assess sarcopenia as described in our previous study.^10^ In brief, the psoas muscle mass index (PMI) was calculated from the psoas muscle area at the level of the third lumbar vertebra (L3) on CT images divided by height squared. Sarcopenia was defined using the previously validated sex‐specific PMI cutoff values for Asian adults of 6.36 cm^2^/m^2^ for males and 3.92 cm^2^/m^2^ for females.^11^

### 2.5 Statistical analysis

All continuous variables are presented as medians and interquartile ranges (IQR). PSM was performed using thirteen factors: age, sex, the American Society of Anesthesiologists‐Physical Status score (ASA‐PS), the Charlson comorbidity index, a history of open surgery, tumor location, the TNM stage, preoperative treatment, surgical approach, type of surgery, stoma creation, combined resection, and lateral pelvic node resection. We performed 1:1 matching between the sarcopenia and nonsarcopenia groups using nearest‐neighbor matching (caliper = 0.2) of the standard deviation of the propensity score logit. The relationships between sarcopenia and clinicopathological variables were analyzed using the chi‐square test for categorical variables and the Mann–Whitney *U* test for nonparametric variables. Kaplan–Meier survival curves and log‐rank tests were used to estimate and compare patient survival. Variables with a *p* value <0.05 in the univariate logistic regression analysis were further examined in multivariate logistic regression analyses to identify predictors of total postoperative complications. All analyses were performed using JMP Pro 15.0 software (SAS Institute, Cary, NC, USA). The *p* values <0.05 were considered significant.

## 3 RESULTS

### 3.1 Clinical characteristics

Among 787 patients, 350 (44.5%) had sarcopenia. The median age of patients who underwent rectal resection was 65 y (IQR, 56–72 y). Table 1 shows baseline characteristics for the entire cohort and matched cases. Before matching, sarcopenia was associated with age, sex, BMI, ASA‐PS, the surgical approach, surgical procedure, and (yp)TNM stage. After matching, no significant differences were observed in any variable, except for BMI. Patients who underwent low anterior resection, abdominal perineal resection, intersphincteric resection, and Hartmann's procedure were included in both groups.

**TABLE 1:** Relationships between baseline characteristics before and after propensity score matching.

	Overall cohort			Matched cohort		
	Sarcopenia	Non‐sarcopenia		Sarcopenia	Non‐sarcopenia	
Variables	(*n* = 350)	(*n* = 437)	*p* Value	(*n* = 266)	(*n* = 266)	*p* Value
Age (years)*	67 (60–74)	62 (54–70)	<0.0001	66 (59–73)	65 (58–73)	0.90
Sex						
Male	253 (72.3)	221 (50.6)	<0.0001	172 (64.7)	161 (60.5)	0.32
Female	97 (27.7)	216 (49.4)		94 (35.3)	105 (39.5)	
BMI (kg/m^2^)*	21.3 (19.1–23.2)	23.0 (21.0–25.4)	<0.0001	21.2 (19.1–23.0)	23.5 (21.2–25.5)	<0.0001
ASA‐PS score						
I	117 (33.4)	188 (43.0)	0.0023	105 (39.5)	106 (39.9)	0.99
II	226 (64.6)	231 (52.9)		155 (58.3)	154 (57.9)	
III	7 (2.0)	18 (4.1)		6 (2.2)	6 (2.2)	
Charlson comorbidity index						
0	228 (65.1)	280 (64.1)	0.76	169 (63.5)	174 (65.4)	0.65
≥1	122 (34.9)	157 (35.9)		97 (36.5)	92 (34.6)	
History of abdominal surgery						
Yes	97 (27.7)	123 (28.1)	0.89	75 (28.2)	82 (30.8)	0.51
No	253 (72.3)	314 (71.9)		191 (71.8)	184 (69.2)	
Preoperative therapy						
Chemotherapy	6 (1.7)	5 (1.1)	0.40	4 (1.5)	4 (1.5)	0.39
Radiotherapy	8 (2.3)	4 (0.9)		7 (2.6)	2 (0.8)	
Chemoradiotherapy	159 (45.4)	199 (45.5)		116 (43.6)	119 (44.7)	
None	177 (50.6)	229 (52.5)		139 (52.3)	141 (53.0)	
Tumor location						
Middle rectum	108 (30.9)	116 (26.5)	0.18	77 (29.0)	85 (32.0)	0.45
Lower rectum	242 (69.1)	321 (73.5)		189 (71.0)	181 (68.0)	
Surgical approach						
Open	104 (29.7)	101 (23.1)	0.036	69 (25.9)	66 (24.8)	0.76
Laparoscopy/Robot	246 (70.3)	336 (76.9)		197 (74.1)	200 (75.2)	
Surgical procedure						
Low anterior resection	241 (68.9)	318 (72.8)	0.0077	188 (70.7)	190 (71.4)	0.97
Abdominal perineal resection	59 (16.9)	56 (12.8)		41 (15.4)	42 (15.8)	
Intersphincteric resection	40 (11.4)	53 (12.1)		33 (12.4)	31 (11.7)	
Total pelvic exenteration	6 (1.7)	0 (0)		0 (0)	0 (0)	
Hartmann's operation	4 (1.1)	10 (2.3)		4 (1.5)	3 (1.1)	
Stoma creation						
Ileostomy	110 (31.4)	160 (36.6)	0.25	94 (35.3)	85 (32.0)	0.70
Colostomy	81 (23.1)	86 (19.7)		52 (19.6)	56 (21.1)	
None	159 (45.4)	191 (43.7)		120 (45.1)	125 (46.9)	
Combined organ resection						
Yes	43 (12.3)	40 (9.2)	0.16	28 (10.5)	25 (9.4)	0.66
No	307 (87.7)	397 (90.9)		238 (89.5)	241 (90.6)	
LPN dissection						
Yes	40 (11.4)	42 (9.6)	0.41	30 (11.3)	29 (10.9)	0.89
No	310 (88.6)	395 (90.4)		236 (88.7)	237 (89.1)	
(yp)TNM stage						
0	19 (5.4)	17 (3.9)	0.066	12 (4.5)	10 (3.8)	0.91
I	104 (29.7)	167 (38.2)		93 (35.0)	96 (36.1)	
II	91 (26.0)	98 (22.4)		66 (24.8)	67 (25.2)	
III	106 (30.3)	131 (30.0)		81 (30.5)	75 (28.2)	
IV	30 (8.6)	24 (5.5)		14 (5.2)	18 (6.7)	
Histopathological type						
Differentiated (Well/Moderate)	326 (93.1)	412 (94.3)	0.51	250 (94.0)	248 (93.2)	0.72
Others	24 (6.9)	25 (5.7)		16 (6.0)	18 (6.8)	
Resected margin						
R0	344 (98.3)	429 (98.2)	0.90	260 (97.7)	263 (98.9)	0.31
R1	6 (1.7)	8 (1.8)		6 (2.3)	3 (1.1)	
R2	0 (0)	0 (0)		0 (0)	0 (0)	
Adjuvant chemotherapy						
Yes	104 (29.7)	122 (27.9)	0.58	78 (29.3)	65 (24.4)	0.20
No	246 (70.3)	315 (72.1)		188 (70.7)	201 (75.6)	

*Note*: Values in parentheses are percentages, unless indicated otherwise.

Abbreviations: ASA‐PS score, American Society of Anesthesiologists‐Physical Status score; BMI, body mass index; LPN, lateral pelvic node; TNM, tumor‐node‐metastasis.

* Values are medians (interquartile ranges).

### 3.2 Short‐term outcomes

Surgical outcomes before and after PSM are summarized in Table 2. After matching, no significant differences were observed in surgical times or estimated blood loss between the groups. The rates of postoperative complications of all grades and CD grade ≥2 were significantly higher in the sarcopenia group (33.1% and 29.3%, respectively) than in the nonsarcopenia group (24.8% and 21.8%, respectively). Regarding CD grade ≥2 postoperative complications, anastomotic leakage occurred only in the sarcopenia group. The incidence of intestinal obstruction and ileus was slightly higher in the sarcopenia group (*p* = 0.062). Severe postoperative complications (CD grade ≥3) occurred in 28 (10.5%) patients in the sarcopenia group and 17 (6.4%) in the nonsarcopenia group; however, this difference was not significant (*p* = 0.085). The rate of reoperation within 30 d after surgery did not significantly differ between the two groups, and no patients died during the study period. The time to first flatus and the length of postoperative stays were similar between the groups.

**TABLE 2:** Perioperative outcomes before and after propensity score matching.

	Overall cohort			Matched cohort		
	Sarcopenia	Nonsarcopenia		Sarcopenia	Nonsarcopenia	
Variables	(*n* = 350)	(*n* = 437)	*p* value	(*n* = 266)	(*n* = 266)	*p* value
Operative time (min)^a^	333 (248–443)	325 (239–437)	0.78	325 (246–438)	325 (235–428)	0.71
Estimated blood loss (mL)^a^	100 (10–340)	110 (19–450)	0.18	100 (10–390)	110 (19–383)	0.96
Postoperative complications (All grades)	111 (31.7)	106 (24.3)	0.020	88 (33.1)	66 (24.8)	0.035
Postoperative complications (CD grade ≥2)						
All	99 (28.3)	95 (21.7)	0.035	78 (29.3)	58 (21.8)	0.047
Any infection	57 (16.3)	55 (12.6)	0.14	43 (16.2)	34 (12.8)	0.27
Surgical site infection (superficial)	7 (2.0)	5 (1.1)	0.33	5 (1.9)	5 (1.9)	1.00
Surgical site infection (Deep/Organ)	31 (8.9)	25 (5.7)	0.090	24 (9.0)	15 (5.6)	0.13
Anastomotic leakage	8 (2.3)	0 (0)	0.0003	5 (1.9)	0 (0)	0.0082
Intestinal obstruction/ileus	19 (5.4)	20 (4.6)	0.59	17 (6.4)	8 (3.0)	0.062
Urination disorder	13 (3.7)	14 (3.2)	0.70	11 (4.1)	11 (4.1)	1.00
Others	19 (5.4)	14 (3.2)	0.12	14 (5.3)	8 (3.0)	0.19
Postoperative complications (CD grade ≥3)	37 (10.6)	29 (6.6)	0.049	28 (10.5)	17 (6.4)	0.085
Reoperation within 30 d after surgery	7 (2.0)	4 (0.9)	0.20	5 (1.9)	2 (0.8)	0.25
Readmission within 30 d after discharge	7 (2.0)	6 (1.4)	0.49	6 (2.3)	3 (1.1)	0.31
Mortality within 30 d after surgery	0 (0)	0 (0)	‐	0 (0)	0 (0)	‐
Time to first flatus (d)^a^	2 (1–3)	2 (1–3)	0.99	2 (1–3)	2 (1–3)	0.39
Length of postoperative stay (d)^a^	19 (15–24)	18 (15–23)	0.087	18 (15–24)	18 (15–22)	0.13

*Note*: Values in parentheses are percentages, unless indicated otherwise.

Abbreviations: CD grade, Clavien–Dindo classification grade.

^a^ Values are medians (interquartile ranges).

### 3.3 Analysis of risk factors for total postoperative complications

Table 3 shows univariate and multivariate analyses of factors associated with total postoperative complications in all cohorts. The multivariate logistic regression analysis identified sarcopenia [*p* = 0.042; odds ratio [OR], 1.41; 95% confidence interval [CI] 1.01–1.96], preoperative CRT/RT/NAC (*p* < 0.0001; OR, 0.42; 95% CI 0.29–0.62), abdominal perineal resection (*p* = 0.036; OR, 2.37; 95% CI 1.06–5.31), Hartmann's operation (*p* = 0.049; OR, 3.71; 95% CI 1.01–13.6), ileostomy creation (*p* = 0.024; OR, 1.67; 95% CI 1.08–2.63), and lateral pelvic lymph node dissection (*p* = 0.014; OR, 1.95; 95% CI 1.14–3.32) as independent risk factors for overall complications.

**TABLE 3:** Univariate and multivariate analyses of risk factors for total postoperative complications.

	Univariate analysis			Multivariate analysis		
	Total (*n* = 787)	Complications (%)	*p* value	OR	95% CI	*p* value
Age (y)			0.29			
<75	646	173 (26.8)				
≥75	141	44 (31.2)				
Sex			0.17			
Female	313	78 (24.9)				
Male	474	139 (29.3)				
Charlson comorbidity index			0.13			
0	508	149 (29.3)				
≥1	279	68 (24.4)				
ASA‐PS score			0.031			
I	305	71 (23.3)		Ref.		
II/III	482	146 (30.3)		1.20	0.85–1.69	0.31
BMI			0.51			
<25	618	167 (27.0)				
≥25	169	50 (29.6)				
Preoperative CRT/RT/NAC			0.025			
No	406	126 (31.0)		Ref.		
Yes	381	91 (23.9)		0.42	0.29–0.62	<0.0001
History of abdominal surgery			0.68			
No	567	154 (27.2)				
Yes	220	63 (28.6)				
Tumor location			0.62			
Middle rectum	224	59 (26.3)				
Lower rectum	563	158 (28.1)				
Surgical approach			0.088			
Open	205	66 (32.2)				
Laparoscopy/Robot	582	151 (26.0)				
Surgical time (min)			0.0006			
<360	469	108 (23.0)		Ref.		
≥360	318	109 (34.3)		1.36	0.92–2.00	0.12
Surgical procedure			0.0017			
Low anterior resection	559	136 (24.3)		Ref		
Abdominal perineal resection	115	45 (39.1)		2.37	1.06–5.31	0.036
Intersphincteric resection	93	25 (26.9)		0.84	048–1.48	0.55
Total pelvic exenteration	6	4 (66.7)		5.10	0.81–32.2	0.083
Hartmann's operation	14	7 (50.0)		3.71	1.01–13.6	0.049
Stoma creation			0.0009			
None	350	75 (21.4)		Ref		
Ileostomy	270	81 (30.0)		1.67	1.07–2.63	0.024
Colostomy	167	61 (36.5)		1.23	0.57–2.68	0.60
Combined organ resection			0.011			
No	704	184 (26.1)		Ref		
Yes	83	33 (39.8)		1.49	0.88–2.52	0.14
LPN dissection			0.0041			
No	705	183 (26.0)		Ref.		
Yes	82	34 (41.5)		1.95	1.14–3.32	0.014
(yp)TNM stage			0.11			
0	36	4 (11.1)				
I	271	76 (28.0)				
II	189	49 (25.9)				
III	237	70 (29.5)				
IV	54	18 (33.3)				
Sarcopenia			0.020			
No	437	106 (24.3)		Ref.		
Yes	350	111 (31.7)		1.41	1.01–1.96	0.042

*Note*: Values in parentheses are percentages, unless indicated otherwise.

Abbreviations: ASA‐PS score, American Society of Anesthesiologists‐Physical Status Score; BMI, body mass index; CI, confidence interval; CRT, chemoradiotherapy; LPN, lateral pelvic node; NAC, neoadjuvant chemotherapy; OR, odds ratio; RT, radiotherapy; TNM, tumor‐node‐metastasis.

### 3.4 Long‐term outcomes after PSM

**FIGURE 2:**
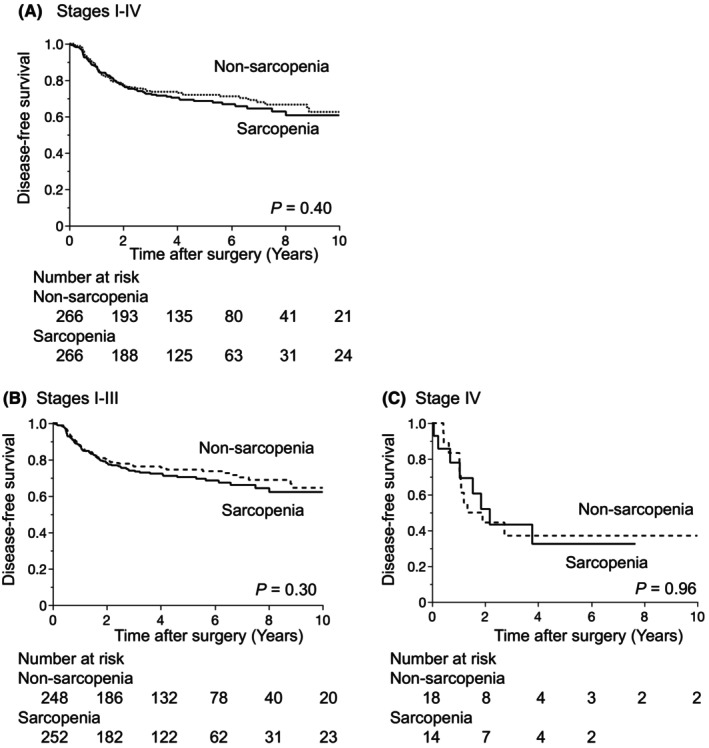
Relationships between the sarcopenic status and disease‐free survival (DFS). DFS curves for Stages I–IV patients (A), Stages I–III patients (B), and Stage IV patients (C).

**FIGURE 3:**
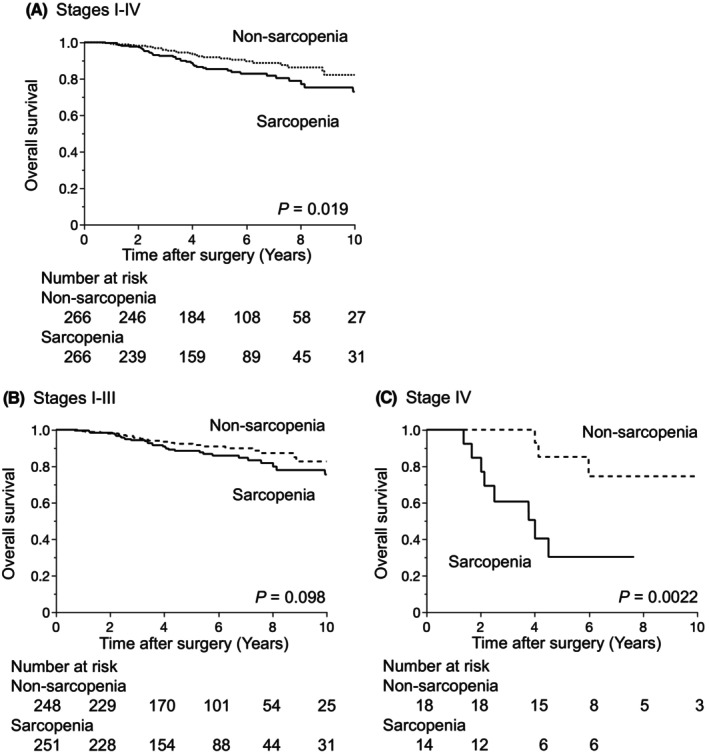
Relationships between the sarcopenic status and overall survival (OS). OS curves for Stages I–IV patients (A), Stages I–III patients (B), and Stage IV patients (C).

The median follow‐up period was 5.03 y, and the 5‐y DFS and OS rates were 70.3% and 88.6%, respectively. DFS was similar between the two groups (*p* = 0.40, Figure 2A). We also investigated the effects of the sarcopenic status on prognosis according to stages without distant metastasis (Stages I–III) and with distant metastasis (Stage IV). The results obtained showed that the sarcopenic status did not affect DFS in Stages I–III or Stage IV (Figure 2B,C). In contrast, OS was significantly worse in the sarcopenia group. Five‐y OS rates in the sarcopenia and nonsarcopenia groups were 85.3% and 91.8%, respectively (*p* = 0.019, Figure 3A). According to the presence of distant metastasis, sarcopenia affected OS in Stages I–III and Stage IV. However, the difference was not significant in Stages I–III (Figure 3B,C).

Based on the differences observed in treatment strategies between Stages I–III and Stage IV rectal cancer, we investigated the relationship between preoperative treatment and long‐term outcomes. In the present study, patients with Stages II/III and Stage IV rectal cancer were more likely to receive preoperative chemoradiotherapy (or radiotherapy) and chemotherapy, respectively. DFS and OS curves were discriminated by preoperative treatment (Figure S1A,B).

## 4 DISCUSSION

The present results demonstrated that preoperative sarcopenia significantly increased the rates of total postoperative complications and anastomotic leakage after PSM. Moreover, preoperative sarcopenia was identified as an independent risk factor for total postoperative complications. OS rates were also significantly lower in the sarcopenia group than in the nonsarcopenia group after PSM. To the best of our knowledge, this is the first study to investigate short‐term outcomes associated with sarcopenia in rectal cancer patients using PSM.

Although recent meta‐analyses reported that a low skeletal muscle mass was associated with short‐term outcomes, such as total postoperative complications, postoperative infections, and a prolonged hospital stay after colorectal cancer surgery, the findings obtained on rectal cancer surgery remain controversial. Regarding postoperative complications in rectal cancer patients undergoing curative resection, previous studies confirmed that age, the distance of the tumor from the anus, and open surgery were risk factors for total postoperative complications.^7^, ^12^ Several factors regarding surgical procedures warrant more attention in rectal cancer surgery than in colon cancer surgery. Procedures beyond TME alone, such as LPND and multivisceral excision, demand higher technical surgical skills, resulting in longer operation times, more blood loss, and postoperative complications. In addition, more patients receive preoperative CRT or radiotherapy for rectal cancer than for colon cancer. Since ~50% of patients who received radiotherapy in the present study and preoperative treatment has been suggested to affect short‐term outcomes, this difference needs to be considered when investigating the effects of sarcopenia independent of surgery. Therefore, we performed PSM in an attempt to mitigate these confounding factors.

The results obtained herein demonstrated that sarcopenia was associated with an increased rate of postoperative complications, which is consistent with previous findings on rectal cancer.^4^ The relationship between sarcopenia and postoperative complications currently remains unclear. Reisinger et al^13^ showed that a low muscle mass was associated with an increased postoperative inflammatory response, resulting in postoperative complications in colorectal cancer surgery. Furthermore, Huang et al^14^ suggested low physical performance related to CD grade ≥2 postoperative complications. Although high‐quality evidence is limited for sarcopenic patients, prehabilitation, including supplemental nutrients and physical interventions, may contribute to the prevention of postoperative complications.^15^

A relationship was previously reported between a low skeletal muscle mass and anastomotic leakage in sigmoid and rectal cancer surgeries,^16^ which is in agreement with the present results. Sarcopenia is associated with metabolic abnormalities, which cause an inflammatory state that may impair normal tissue repair and anastomotic healing.^17^, ^18^, ^19^ Based on meta‐analyses, sarcopenia was identified as a risk factor for anastomotic leakage after esophagectomy^20^; however, previous studies found no correlation between sarcopenia and anastomotic leakage in colorectal cancer^3^ or gastric cancer surgery.^21^ Since the incidence of anastomotic leakage is higher in rectal cancer surgery than in colon cancer surgery, further studies are needed to clarify the relationship between sarcopenia and anastomotic leakage in rectal cancer surgery.

The rate of postoperative ileus was previously shown to be significantly higher in patients with preoperative sarcopenia after colorectal cancer^22^ and rectal cancer^23^ surgeries, which is consistent with the present results. Traeger et al proposed that sarcopenic patients are more likely to have a nutritional imbalance, leading to a proinflammatory state, resulting in postoperative ileus.^22^ In addition, changes were recently detected in the gut microbiome of older adults with low handgrip strength.^24^ The intestinal flora may affect bowel function, leading to ileus or an imbalance in intestinal fluid absorption after colorectal surgery. A high‐output volume after ileostomy creation has been shown to cause stoma outlet obstruction,^25^ which may explain the relationship between sarcopenia and postoperative ileus and obstruction.

Although sarcopenia significantly increased the rates of postoperative complications of all grades and CD grade ≥2, no significant differences were observed in the length of hospital stays after surgery. One reason may be this study's high rate of stoma creation, more than 50%, which is different from colon cancer surgery. Generally, patients who underwent stoma creation require at least an extra 1 or 2 weeks of hospital stays to learn stoma care management with or without postoperative complications.

The multivariate analysis of our cohort identified the surgical procedure, ileostomy creation, and LPN dissection as independent risk factors for postoperative complications. The impact of these factors on postoperative complications was demonstrated in previous studies. Several surgical procedures have been shown to increase the rate of postoperative complications. High rates of both perineal wound complications^26^ and perineal hernia^27^ have been reported following abdominal perineal resection. Furthermore, Hartmann's operation has been associated with significantly more minor complications than abdominal perineal resection.^28^ This procedure is often selected for emergency settings or patients with comorbidities, leading to worse short‐term outcomes.^29^ While LPN dissection aims to reduce local recurrence and improve long‐term outcomes, it increases overall postoperative complications.^30^ Additionally, a previous meta‐analysis suggested that diverting ileostomy creation protected against anastomotic leakage in patients undergoing rectal resection. However, diverting ileostomy creation was found to increase postoperative complications more than anastomotic leakage.^31^ On the other hand, preoperative treatment was an independent favorable factor for postoperative complications. This result was unexpected, due to the lack of an established relationship between preoperative treatment and postoperative complications in rectal surgery,^32^ and may be partly attributed to expert surgeons at our institution frequently performing rectal surgery on challenging cases. This potential selection bias represents a limitation of our study. The inherent limitation of our single‐center retrospective design, along with the surgical factors affecting complication rates, strengthens the rationale for employing a PSM analysis in this study to mitigate potential confounders.

Regarding long‐term outcomes after matching, OS, but not DFS, significantly differed between the sarcopenia and nonsarcopenia groups in the present study, which is consistent with previous findings, including our studies on a different cohort.^33^ We also investigated the impact of the sarcopenic status on Stage IV disease due to differences in perioperative treatment strategies and prognosis between Stage IV and Stages I–III. Survival curves after propensity score matching may be more meaningful due to the mitigation of these confounders. Our analysis revealed that sarcopenia significantly affected OS, but not DFS, in patients with Stage IV rectal cancer. This result is consistent with previous findings.^34^ Previous studies demonstrated that colorectal patients with sarcopenia were less tolerant of chemotherapy than those without sarcopenia.^35^ Therefore, worse survival outcomes in the sarcopenia group than in the nonsarcopenia group may be attributed to dose reductions and fewer cycles of chemotherapy. Since the rate of recurrence was higher in patients with rectal and Stage IV cancers than in those with colon cancer, sarcopenia may have a stronger impact on treatment responses or tolerability after recurrence than recurrence in patients with an aggressive malignant status.

There are a number of limitations that need to be addressed. The present study was a single‐center retrospective cohort study. Since the optimal cutoff value for sarcopenia remains unclear in the oncology field, further studies are warranted. Furthermore, radiological anastomotic leakage lacking clinical symptoms was possibly underestimated because a postoperative contrast enema assessment was not routinely performed. In the present study, baseline characteristics significantly differed between the sarcopenia and nonsarcopenia groups and PSM was applied to eliminate bias and confounding factors. However, several variables, such as BMI and the nutritional status, were not excluded. In addition, unknown confounding factors may not have been entirely excluded.

In conclusion, we demonstrated that preoperative sarcopenia was associated with more total postoperative complications, more anastomotic leakage, and worse survival in rectal cancer patients.

## AUTHOR CONTRIBUTIONS

**Shinya Abe:** Conceptualization; data curation; formal analysis; investigation; methodology; project administration; visualization; writing – original draft; writing – review and editing. **Hiroaki Nozawa:** Data curation; writing – review and editing. **Kazuhito Sasaki:** Data curation; writing – review and editing. **Koji Murono:** Data curation; writing – review and editing. **Shigenobu Emoto:** Data curation; writing – review and editing. **Kensuke Kaneko:** Data curation; writing – review and editing. **Yuichiro Yokoyama:** Data curation; writing – review and editing. **Hiroyuki Matsuzaki:** Data curation; writing – review and editing. **Yuzo Nagai:** Data curation; writing – review and editing. **Soichiro Ishihara:** Conceptualization; project administration; supervision; writing – review and editing.

## FUNDING INFORMATION

This research was supported by Japan Society for the promotion of Science (B: grant number; 21H02778) from the Japan Society for the Promotion of Science.

## CONFLICT OF INTEREST STATEMENT

Drs. SA, HN, KS, KM, SE, KK, YY, HM, YN, and SI have no conflicts of interest or financial ties to disclose.

## ETHICS STATEMENT

Approval of the research protocol was by an Institutional Reviewer Board: The present study was approved by the Institutional Ethics Committee of The University of Tokyo (No. 3252‐[15]).

Informed Consent: Informed consent was obtained through an opt‐out method.

Registry and the Registration No. of the study/Trial: NA.

Animal Studies: NA.

## Supporting information


**Figure S1:** Relationships between the preoperative treatment strategies and disease‐free survival (DFS) and overall survival (OS). Kaplan–Meier DFS (A) and OS (B) were stratified according to the preoperative treatment strategies.
