# Supplementary material for: Preoperative sarcopenia negatively impacts short‐ and long‐term outcomes of rectal cancer: A propensity score‐matched analysis
Source: Ann Gastroenterol Surg. 2024 Nov 24;9(3):518–28. doi: 10.1002/ags3.12889 (PMC12080190; doi:10.1002/ags3.12889)
Supplement: Supplementary file 1 — Figure S1: Relationships between the preoperative treatment strategies and disease‐free survival (DFS) and overall survival (OS). Kaplan–Meier DFS (A) and OS (B) were stratified according to the preoperative treatment strategies. [file AGS3-9-518-s001.pptx]

## Slide 1
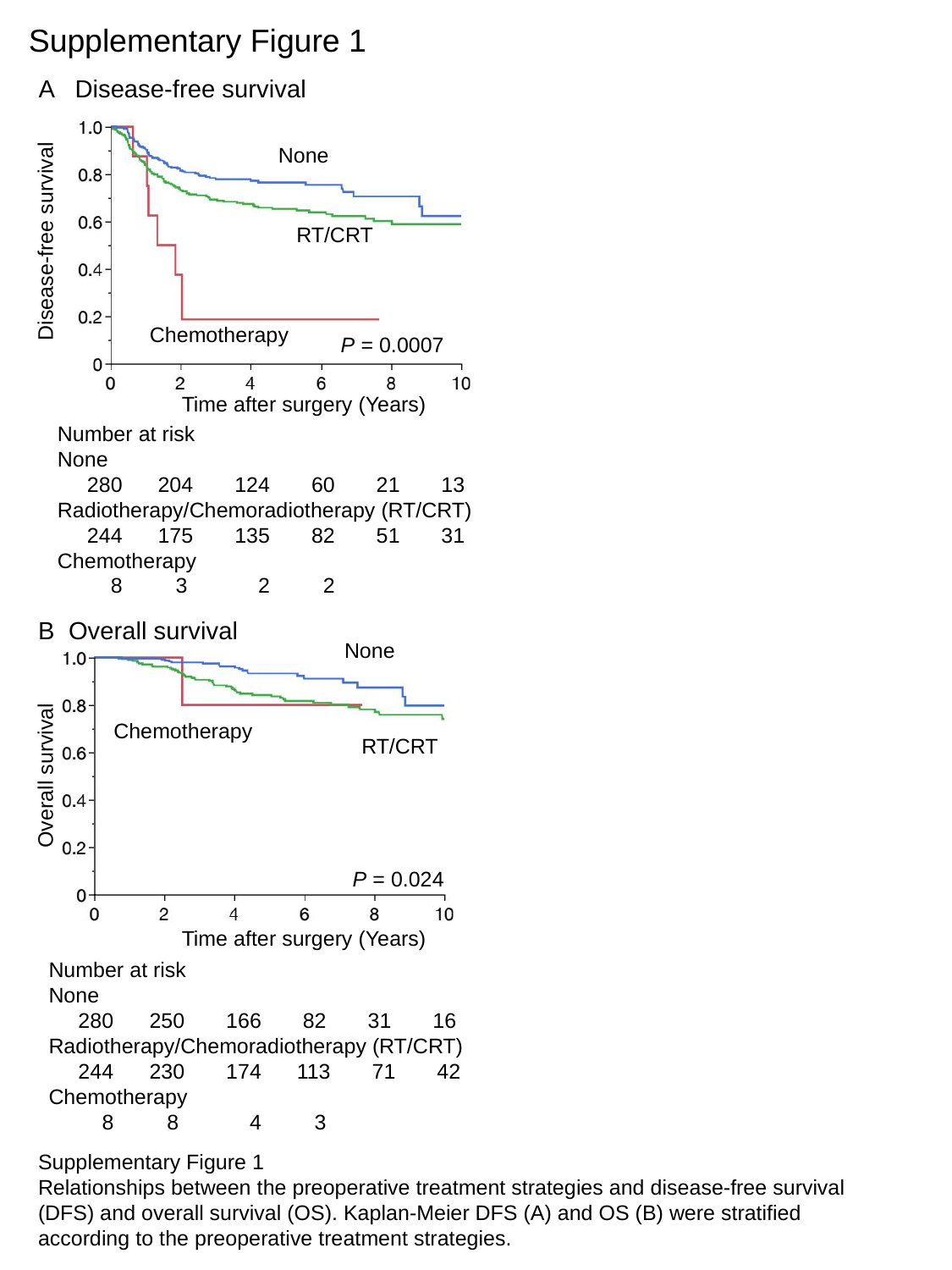

Supplementary Figure 1
A Disease-free survival
None
RT/CRT
Disease-free survival
Chemotherapy
P = 0.0007
Time after surgery (Years)
Number at risk
None
 280 204 124 60 21 13
Radiotherapy/Chemoradiotherapy (RT/CRT)
 244 175 135 82 51 31
Chemotherapy
 8 3 2 2
B Overall survival
None
Chemotherapy
RT/CRT
Overall survival
P = 0.024
Time after surgery (Years)
Number at risk
None
 280 250 166 82 31 16
Radiotherapy/Chemoradiotherapy (RT/CRT)
 244 230 174 113 71 42
Chemotherapy
 8 8 4 3
Supplementary Figure 1
Relationships between the preoperative treatment strategies and disease-free survival (DFS) and overall survival (OS). Kaplan-Meier DFS (A) and OS (B) were stratified according to the preoperative treatment strategies.
